# Supplementary material for: Are carbon emissions trading and green financial instruments synergistic? -Comprehensive quantitative research based on content analysis
Source: PLoS One. 2024 Mar 7;19(3):e0298601. doi: 10.1371/journal.pone.0298601 (PMC10919601; doi:10.1371/journal.pone.0298601)
Supplement: S1 Data — (ZIP) [file pone.0298601.s001.zip › Data/地级市Z_F工作报告.zip!/╦─┤¿/─╧│Σ╩╨/└┤╫╘╣1⁄2╓┌║┼í╛┬φ┐╦╩2╛▌═°í┐_2016.docx]

各位代表：

　　我代表市人民政府向大会报告工作，请予审查，并请市政协委员和其他列席会议的同志提出意见。

一、“十二五”时期发展主要成就和2015年工作回顾

　　“十二五”时期，是南充发展史上极不平凡的五年。五年来，面对多重困难叠加、多重任务交织的考验，我们认真贯彻省委省政府和市委的决策部署，坚持稳中求进的工作总基调，积极主动适应经济新常态，始终保持专注发展定力，在攻坚克难中激流勇进，在开拓创新中爬坡越坎，顺利完成“十二五”规划确定的目标任务，经济社会发展取得新的历史性成效。

　　五年来，我们着力加快发展、追赶跨越，综合实力大幅提升。经济总量稳居全省前列、川东北首位，2011年在全省地级市中率先迈进“千亿元俱乐部”，2015年突破1500亿元，年均增长11%。全社会固定资产投资、一般公共预算收入实现“翻番”，年均分别增长16.2%、21.4%。规模以上工业增加值年均增长13.7%。社会消费品零售总额年均增长15.4%。城镇居民人均可支配收入与全省平均水平的差距从2823元缩小到2255元，农村居民人均可支配收入超过全省平均水平。全市人民币各项存款余额2561亿元、贷款余额1294亿元，年均分别增长18.4%、25%。县域经济实力得到增强，9县（市、区）地区生产总值均超过百亿元，顺庆区、南部县突破250亿元，阆中市、仪陇县达到150亿元以上。

　　五年来，我们着力产业支撑、结构优化，实体经济不断壮大。工业开始向集群化发展、中高端迈进，以南充经开区、南充新能源汽车产业园为龙头的“2+9”工业园区建设扎实推进，全市园区建成面积达94平方公里、主营业务收入突破1500亿元；“4+3”特色优势产业进一步做大做强，2015年，规上工业主营业务收入达到2100亿元；一批重大工业项目加快推进，吉利南充新能源商用车研发生产、中国重汽南充生产基地等项目启动建设，晟达新材料、鑫达新材料等项目即将建成投产。农业产业园区建设和特色产业基地培育取得新进展，南充国家现代农业示范区在全国农业改革与建设试点示范区绩效考评中连续两年获西部第一，成功申报国家农业科技园区，启动建设中法农业科技园；建成农业特色优势产业基地350万亩，全市认证有机农产品基地17.8万亩，西充被确定为国家有机产品认证示范县；升钟水库灌区二期、油房沟水库和金鸡沟水库建设加快推进，解元水库、九龙潭水库全面竣工；粮食总产量连续13年居全省第一。服务业加快发展，成功申报全省服务业中心城市和全国首批信息消费试点城市；南部、营山亿联商贸城和阆中义乌小商品城等商业综合体建成投入营运；金融机构快速聚集，总量居全省第二；成功举办中国四川国际文化旅游节、四川省第六届乡村文化旅游节，阆中古城创建为国家5A级旅游景区。

　　五年来，我们着力夯实基础、枢纽打造，发展条件明显改善。立体交通网络基本形成，兰渝铁路南充段建成通车，境内新增铁路里程216公里、总里程达到334公里，汉巴南铁路、蓉京高铁（成南达段）和遂南广邻城际铁路纳入《四川省铁路网规划》。新建成高速公路6条，总条数达到10条、总里程461公里，实现县县通高速。高坪机场改扩建工程顺利完工，航站区改扩建工程加快推进，年旅客吞吐量达到55万人次。南充港都京作业区多用途码头、旅游客运码头和南充经开区专用码头开港营运，嘉陵江航运配套工程扎实推进，通江达海的“黄金水道”初步形成。区域物流中心加快建设，成功申报全国区域流通节点城市、全省首批现代物流试点示范市，南充现代物流园开园营运。通讯信息枢纽加快完善，区域网络和通信基础设施实现升级扩容，“三网融合”有序推进，浪潮集团大数据处理中心加快建设，城区及农村所有场镇实现光纤全覆盖，在川东北的综合通信能力领先优势进一步扩大。

　　五年来，我们着力功能完善、品质提升，城乡建设统筹推进。全市城镇化率达到43.8%，年均增长1.6个百分点。城镇功能不断完善，中心城区北部新城、南部产业新城和清泉坝、下中坝等城市新区加快建设，嘉陵江四桥、西河路南延线、西河体育公园等建成投入使用，建成区面积达到115.3平方公里，城市人口达到110万人；县级城市承载能力和吸纳能力进一步增强，阆中入选国家新型城镇化综合试点县（市），南部获批国家中小城市综合改革试点县，西充被纳入全省全域规划试点县；23个国省重点（试点）小城镇、9个市级扩权镇建设加快推进，672个幸福美丽新村、1701个新村聚居点基本建成。生态建设得到加强，我市被纳入全国第二批生态文明先行示范区建设范围，阆中成功创建国家园林城市，西充成功创建全国绿化模范县，全市森林覆盖率达到39.8%；狠抓总量减排和重点流域、危险废物、重金属污染防治，嘉陵江干流水质稳定达标，西充河流域水质明显改善，单位GDP能耗累计下降20.75%。城市管理水平得到提高，市辖城区数字城管系统建成投入使用；城乡环境综合治理成效明显，城乡面貌不断改善。

　　五年来，我们着力深化改革、扩大开放，发展活力逐步释放。重点领域改革不断深化，农村产权制度改革基本完成集体土地所有权、集体建设用地使用权确权颁证，全面完成林权确权颁证，9县（市、区）农村产权流转交易中心全部挂牌成立。农信社改制步伐加快，仪陇、营山、西充、阆中4家农商行挂牌开业。国资国企改革力度加大，东风南充汽车公司国有股权挂牌转让，康源水务公司实现市区水务一体化。财税体制改革稳妥推进，与事权相匹配的市区财权管理体制进一步完善。公立医院改革进展顺利，药品加成全面取消，分级诊疗制度有效实施，川北医学院附属医院集团和市医疗集团的影响力、辐射力进一步增强，成功申报第二批国家公立医院改革联系试点城市。行政审批制度改革不断深化，非行政许可事项全面取消。对外交流合作成效明显，成功举办四川（南充）·法国周、首届中国南充国际木偶艺术周等活动，与欧美国家的合作进一步深化；坚持集群招商、沿链引进，累计到位资金2098.6亿元，10家世界500强、28家国内500强、9家大型央企、44家上市公司落户南充。

　　五年来，我们着力改善民生、共建共享，群众福祉持续增强。强化公共财政民生投向，财政用于民生投入累计达到1045亿元。脱贫攻坚步伐加快，54.58万农村贫困人口脱贫解困。新增城镇就业年均超过7万人次，城镇登记失业率控制在4.2%以内。基本公共服务得到加强，全市新建、改（扩）建城乡学校（幼儿园）767所，顺庆、阆中、仪陇建成国家义务教育发展基本均衡县，西华师范大学科技园成功创建川东北地区首家省级大学科技园；市中心医院门诊住院大楼、川北医学院附属医院新院区、市中医院综合楼和24家县级医院、267个乡镇卫生院、1536个村卫生室建成投入使用；成功举办中国（南充）嘉陵江合唱艺术节、中国（南充）嘉陵江灯戏暨地方戏剧艺术节、中国升钟湖钓鱼大赛等国际性、全国性文化体育活动，国家公共文化服务体系示范区创建扎实推进，市、县、乡、村公共文化服务网络实现全覆盖。社会保障体系不断完善，实现城乡居民养老保险制度整合和医疗、工伤、生育保险市级统筹，新型农村合作医疗参合率达到98%以上，城乡居民大病医疗保险试点和城镇补充医疗保险工作有序推进；社会救助体系更加健全。累计建成保障性住房和棚户区改造安置住房21.5万套，改造农村危房16.7万户。新（改）建农村公路11098公里，安装公路安保设施4032公里，解决210万农村人口和43万学校师生安全饮水问题。“平安南充”创建活动深入开展，全市无重特大食品药品安全事故发生，安全生产和社会治安形势持续好转，社会大局和谐稳定。

　　五年来，我们着力转变职能、践行法治，自身建设切实加强。扎实开展党的群众路线教育实践活动和“三严三实”专题教育，突出问题导向，狠抓问题整改，着力树立为民务实清廉形象。坚持民主施政，在全省率先建立市、县两级政务微博发布厅和市政府官方微信，在全国率先探索建立政务公开标准化体系。坚持务实勤政，全面推行政府部门绩效管理，持续开展社会满意度测评，挂图作战推进重点项目、重大决策落实落地。坚持高效理政，不断优化行政审批流程，压缩审批时间，行政审批效能进一步提高；省、市、县三级联网运行系统全面建成，市、县、乡三级政务服务网加快建设，“12345”市民服务热线开通运行。坚持依法行政，自觉接受市人大及其常委会的法律监督、工作监督和市政协的民主监督，建立市级领导“一对一”法律顾问制度，严格规范行政执法行为，切实将各项政府工作纳入法治化轨道。坚持廉洁从政，严格执行中央“八项规定”和省委省政府“十项规定”，持续用力正风肃纪，“四风”问题得到有效遏制；深刻汲取南充拉票贿选案惨痛教训，以刮骨疗毒的决心狠抓整改，政治生态和社会风气得到好转。

　　与此同时，国防动员、人民防空和双拥优抚工作扎实推进，行政监察、审计监督、国资监管、统计调查、检验检疫、工商、质监、海关、气象、档案、保密、国安、邮政、外事侨务对台等工作取得新成绩，民族宗教、地方志、老龄、妇女儿童、青少年、残疾人、红十字会等工作发挥了积极作用。

　　各位代表，2015年，是南充发展史上极其特殊、极为艰难的一年。面对严峻复杂的国内外形势和持续加大的经济下行压力，在省委省政府和市委的坚强领导下，全市上下以崛起危难的勇气和浴火重生的决心，下大功夫调结构转方式，下大力气补短板惠民生，经济社会持续健康发展。全年实现地区生产总值1516.2亿元、增长7.6%，完成全社会固定资产投资1389亿元、增长11.6%，社会消费品零售总额698.8亿元、增长12%，规模以上工业增加值增长9.8%，一般公共预算收入85.1亿元、增长7.8%，城镇居民人均可支配收入23950元、增长8.5%，农村居民人均可支配收入10292元、增长10%。

　　各位代表！过去五年，南充经受了经济增速换挡、产业转型升级等发展阵痛，克服了资源要素趋紧、社会风险加剧等现实困难，谱写了开拓奋进、追赶跨越的崭新篇章。这些成绩的取得来之不易，饱含着全市干部群众的辛劳与付出，凝聚着社会各界人士的智慧和力量。在此，我代表市人民政府，向全市各族人民，向各民主党派、工商联、无党派人士和人民团体，向驻市解放军、武警部队官兵、政法干警和各界朋友表示衷心的感谢，并致以崇高的敬意！

　　同时，我们也清醒地认识到，经济社会发展还存在不少困难和问题：总量小、人均少、欠发达的市情还没有根本改变；产业结构性矛盾依然突出，创新能力严重不足，新的增长动力尚处于培育阶段；城乡区域发展还不平衡，农村贫困人口量大面宽，同步全面小康任务十分艰巨；社会事业和民生领域欠账较多，公共服务均等化水平有待提升；政府自身建设任重道远，依法行政水平和政务服务效能亟待提高。对于这些问题，我们一定高度重视，有针对性地采取措施，下大力气切实有效加以解决。

二、“十三五”时期发展的主要考虑

　　根据《中共南充市委关于制定国民经济和社会发展第十三个五年规划的建议》，市政府编制了《南充市国民经济和社会发展第十三个五年规划纲要（草案）》提交大会审查。《纲要（草案）》紧紧围绕全面建成小康社会的奋斗目标和建设区域中心城市的发展定位，强调牢固树立并贯彻落实创新、协调、绿色、开放、共享的发展理念，明确了今后五年南充发展的主要目标、主要抓手和主要路径。我们必须以新理念推动新发展，以新举措开创新局面，共同谱写南充“十三五”发展的宏伟篇章。

　　（一）关于发展的主要目标。按照市委确立的建设创新、开放、生态、法治、繁荣的区域中心城市的发展定位，紧紧围绕与全国全省同步全面小康的战略任务，立足我市发展的阶段性特征和未来发展的支撑条件，挖掘潜力，释放活力，提升竞争力，努力实现“五个走在区域前列”的主要目标，即：全市地区生产总值保持高于全国、快于全省的增速，实现经济强市建设走在区域前列；重要领域关键环节改革取得决定性成果，创新型城市和内陆开放型经济战略高地建设加快推进，实现改革创新和扩大开放走在区域前列；着力创建国家生态市和国家生态文明先行示范区，嘉陵江中游生态屏障基本建成，实现生态绿色发展走在区域前列；传承弘扬“勤劳、朴实、诚信、包容”的城市精神，积极创建全国卫生城市和全国文明城市，实现全民文明素质提升走在区域前列；城乡居民人均可支配收入持续增长，现行标准下贫困人口全面脱贫、贫困县全部摘帽，教育、卫生、文化、体育等社会事业全面发展，实现人民生活质量改善走在区域前列。

　　（二）关于发展的主要抓手。实现“十三五”时期发展目标，必须始终抓住项目这个“牛鼻子”，以项目工作为总抓手，统揽经济社会发展全局，打好项目大会战，实现建设大提速，推动南充大发展。坚持创新思维谋划项目，既立足当前又着眼长远，既放大优势又补齐短板，重点围绕基础设施、产业转型、新型城镇化、民生改善等方面，科学谋划一批基础性强、支撑性强、带动性强的重大项目，力争更多项目进入国省规划“笼子”。坚持全力以赴争引项目，认真研究分析国际国内经济走势，积极对接国家产业政策和投资政策，围绕重点领域、重点产业、重点品牌，积极争取国省重大产业布局，强力实施招大引优、招大引强，为长远发展积蓄能量。坚持挂图作战推进项目，对重大项目列出时间表，制定路线图，用创新的思维、超常的手段强力推进，持续保持投资增长的强劲态势。坚持主动服务保障项目，着力提升政务服务效能，切实加强要素保障，为项目实施营造良好环境。

　　（三）关于发展的主要路径。贯彻落实“五大发展理念”，大力实施富民强市“十大行动”，是建设区域中心城市的主要路径，是保持战略定力的集中体现。我们必须大力实施综合枢纽建设行动，努力构筑区域综合交通、通讯信息、能源和水利枢纽。大力实施产业振兴行动，加快调结构、转方式，做大做优做强实体经济，着力构建优势突出、特色鲜明的现代产业体系。大力实施城乡统筹发展行动，围绕建设山水田园城市，推动区域协同、城乡一体发展。大力实施嘉陵江流域综合保护开发行动，着力打造以嘉陵江为主线的生态走廊和经济走廊。大力实施脱贫攻坚行动，确保“2018年基本脱贫、2019年巩固提升、2020年全面小康”目标如期实现。大力实施民生改善行动，注重机会公平，强化公共服务，扩大有效供给，补齐民生短板，让人民群众共享改革发展成果。大力实施创新创业行动，依靠创新驱动，着力优化资源配置、人才结构和创新创业环境，充分激发全社会创新潜能和创业活力。大力实施重点改革突破行动，切实解决制约经济社会发展的突出问题，用实实在在的成效释放改革红利。大力实施开放合作行动，全面融入“一带一路”和长江经济带建设，努力构建全方位、宽领域、多层次的开放格局。大力实施依法治市行动，加强法治政府建设，自觉把政府工作纳入法治化轨道，自觉把依法行政贯穿于政府工作始终。

三、2016年重点工作

　　2016年，是实施“十三五”规划的开局之年，也是推进结构性改革的攻坚之年。今年政府工作的基本思路是：全面落实党的十八大和十八届三中、四中、五中全会精神，省委十届六次、七次全会和市委五届十次全会精神，深入贯彻习近平总书记系列重要讲话精神，按照“五位一体”总体布局和“四个全面”战略布局，深入实施省委“三大发展战略”和市委富民强市“十大行动”，大力弘扬南充城市精神，统筹推进稳增长、促改革、调结构、惠民生、防风险，着力抓好去产能、去库存、去杠杆、降成本、补短板，为决战决胜全面小康，加快建设区域中心城市奠定坚实基础。

　　全市经济社会发展主要预期目标是：地区生产总值增长7.6%，全社会固定资产投资增长12%，一般公共预算收入增长7.8%，规模以上工业增加值增长9.8%，社会消费品零售总额增长12%，城乡居民人均可支配收入分别增长8.5%、10%，城镇化率提高1.4个百分点。

　　围绕上述目标，重点抓好六个方面工作：

　　（一）突出改革创新，充分激发创新创业活力

　　着力搭建创新创业载体。加快建设创新驱动发展试验区，启动建设创新创业孵化中心和国家农业科技园区孵化中心，扎实推进大学生科技园、中法文化创意产业园建设，力争创建为省级高新技术产业园区。发挥大众创业、万众创新和“互联网+”集众智、汇众力的乘数效应，积极发展众创、众包、众扶、众筹等新模式，支持建设“孵化+创投”、创新工场等新型孵化器。加快建设西华师范大学科技园，支持现有产业园区创建省级高新技术产业园区，鼓励县（市、区）开展省级创新型城市试点。

　　积极培育创新创业主体。加快推进创新改革，鼓励科技成果、知识产权作价出资入股，支持企业知识产权质押融资，扶持科技人员创办科技型企业。支持发展产业技术研究院、工程技术研究中心、产业研发创新战略联盟，加快构建以市场为导向、以企业为主体、产学研用相结合的技术创新体系。深入实施“嘉陵江英才工程”，积极引进领军人才、专业技术人才和创新创业团队，壮大创新创业队伍；大力实施大学生创业引领计划，强化职业教育和技能培训，提升创新创业能力。大力实施“归雁计划”，加强与异地南充商会的深度合作，吸引优秀企业家返乡创业。

　　扎实抓好结构性改革。健全完善支持企业发展的具体措施和有效办法，鼓励企业培育具有市场竞争力的本土品牌，尽力盘活停产半停产企业。推进以满足新市民住房需求为主要出发点的住房制度改革，探索建立购租并举的住房制度，全面实行棚户区改造货币化安置，扩大有效需求，疏通供需渠道，逐步消化房地产市场存量。扎实开展降成本增效益专项行动，全面落实降低实体经济企业成本的政策措施，抓住关键点，打好组合拳，努力降低企业制度性交易成本和生产经营性成本。

　　深入推进重点领域改革。扎实开展农村产权制度改革，全面完成农村产权确权颁证，加快完善农村产权流转交易市场体系。有序推进投融资体制改革，力争实现全市农信社全面改制为农商行，推动南充发展公司、市国投公司、市工投公司等融资平台转型发展，探索成立中小微企业转贷基金公司和农村专业合作资金互助社，力争实现村镇银行县域全覆盖。稳步推进公立医院改革，不断完善管理体制和运行机制，积极引进社会资本参与公立医院发展。着力深化国资国企改革，大力发展混合所有制经济，探索开展企业员工持股试点。

　　（二）强化投资拉动，不断增强经济发展后劲

　　强力抓好基础设施建设。大力实施“南充交通三年推进方案”，开工建设南泸高速公路和南充过境高速公路广南至南广段，加快建设巴南广、绵西和营达高速公路，扎实推进一批国省干线公路改造升级和一级公路、农村公路建设。抓紧推进嘉陵江航运配套工程、南充港都京作业区一期后续工程建设。加快实施高坪机场航站区改扩建工程，逐步开通更多国内航线和一些国际航线；认真做好阆中机场前期工作。加快推进升钟水库灌区二期、金鸡沟水库建设，开工建设双叉河水库，抓好亭子口灌区工程前期工作，确保油房沟水库年内竣工。加快推进“数字南充”“光网南充”和“智慧城市”建设，启动“宽带中国”示范城市和信息惠民试点城市建设。加大农村电网改造力度，完善区域性油气管网建设。

　　务实抓好招商引资工作。抢抓“一带一路”和长江经济带建设战略机遇，依托兰渝铁路过境交汇，主动融入“成渝西昆菱形经济圈”，进一步加强与重庆、西安等毗邻区域的合作，巩固提升与长三角、珠三角、闽三角、环渤海湾及港台等发达地区的联系。积极参加“中外知名企业四川行”“西博会”等重大招商活动，精心组织开展“朱德诞辰130周年”“汽车汽配产业专题招商”等重大活动，扎实有效做好投资促进、经贸合作等工作。加强与海外商（协）会、同乡会及驻海外商务机构的合作，积极拓展境外招商区域，提升对外合作的层次和水平。

　　全力抓好项目包装争取。积极对接国省政策导向和投资方向，精心谋划一批战略性、基础性重大项目，全力争取嘉陵江流域综合保护开发、蓉京高铁（成南达段）、汉巴南铁路、南充新能源汽车产业园、南充创新驱动发展试验区、中法农业科技园等项目进入国省“十三五”专项规划和重点区域规划，力争南充至潼南高速公路、太极集团四川（西充）生产基地等项目年内纳入国省项目库。着力提升项目包装成熟度，丰富项目储备，强化动态管理。

　　（三）加快转型升级，全力促进实体经济振兴

　　狠抓工业提质增效。以实施“强基工程”为抓手，着力打造工业发展平台，抓紧推进南充新能源汽车产业园基础设施建设，力争吉利南充项目年内完成整车生产线厂房建设及设备安装。加快推进南充经开区晟达新材料系列项目建设，确保精对苯二甲酸、工业异辛烷、苯酐及增塑剂等重点项目投产运行。按照“一园一主业、一园一特色”的原则，鼓励9县（市、区）产业园区错位发展，加快建设中国重汽南充生产基地、富安娜现代智能家居等重点项目，确保鑫达新材料、创越炭材料等项目竣工投产。

　　加快发展现代农业。充分发挥南充国家现代农业示范区“试验田”作用，积极推动特色产业基地规模扩张和档次提升，年内新建特色规模种植基地11万亩。突出抓好国家农业科技园区和中法农业科技园区建设，抓紧申报认证有机农产品和有机农产品基地；规划建设区域有机农产品交易平台，打造全国知名的放心农产品生产基地。加快推进农村三次产业互动共融，积极发展大田托管、农产品加工、仓储物流等市场化服务，着力促进农业与休闲旅游、健康养老等产业融合发展。

　　大力发展现代服务业。充实完善南充现代物流园业态，力争启动建设中国物流南充现代物流中心、海尔（南充）虚实网服务园，确保南鑫国际建材物流城和友信龙农产品批发中心建成投产，积极创建国家级示范物流园。适应消费升级趋势，支持发展养老、健康、家政、教育、文化等服务消费。大力实施“促进电子商务快速发展三年行动方案”，加快推进电商产业园、电商网络平台和综合信息平台建设，鼓励线上线下互动，推动实体商业创新转型。全力支持朱德故里景区创建国家5A级旅游景区，大力支持阆中古城创建国家级旅游度假区。

　　（四）着力城乡统筹，努力构建协调发展格局

　　强化中心城区带动作用。加快完善规划体系，科学编制旧城更新、综合管廊、城市亮化等专项规划，启动编制南西蓬一体化发展规划。着力完善城市功能，配套建设清泉坝、下中坝、荆溪片区等城市新区基础设施，加快建设都京港嘉陵江大桥、火车北站配套汽车客运站等项目，大力实施地下综合管廊工程，稳步提高综合承载能力。努力提升城市品位，启动清泉坝“海绵城市”建设试点，抓紧实施滨江南路综合改造和西充河生态旅游景观长廊建设，不断美化城市生态空间。着力加强城市管理，紧紧围绕“摆顺、扫干净”，切实加大治脏、治乱、治堵力度，加快建设智能交通管理系统，完善县（市、区）数字城管系统，建立以社区为单元的网格化管理机制，不断提高城市管理精细化、智能化水平。

　　提升城镇宜居宜业水平。积极抢抓试点机遇，扎实推进阆中国家新型城镇化综合试点、南部国家中小城市综合改革试点。稳步推进协同发展，力争南部撤县设市、西充撤县设区实现突破，启动蓬安撤县设区相关工作。按照精明增长、紧凑城市的理念，推进县城有序拓展规模，完善配套功能，着力增强县城对人口、产业的吸纳能力和承载能力。加快推进国省重点（试点）小城镇和市级扩权镇建设，建成一批综合实力强、辐射范围广的品牌城镇。按照现代生活标准、农村生活形态的思路，启动建设幸福美丽新村500个，新建、保护、改造新村聚居点680个。

　　加强生态安全屏障建设。扎实推进嘉陵江流域综合保护开发，加快编制流域综合保护开发利用总体规划和空间布局规划，以及沿江生态保护、旅游发展、城镇建设等各类专项规划；大力开展沿江绿化，加快建设嘉陵江南充段绿色生态走廊。大力实施天然林保护、退耕还林、水土保持等生态工程，积极创建国家生态市、国家生态文明先行示范区。严格执行新修订的《环境保护法》，扎实开展重点工业污染源、规模化畜禽养殖限期治理，切实加强大气污染防治，深入实施城乡净化行动，全面完成省政府下达的环境质量和总量减排目标。

　　（五）坚持精准扶贫，加快推进脱贫攻坚进程

　　认真开展精准识贫。扎实开展精准识别建档立卡“回头看”，严谨细致开展贫困人口信息数据比对、排查和核实，全面完成贫困户基本信息入库造册上网，实现联网运行，接受公众监督。强化动态管理，开展定期核查，实现扶贫台账与脱贫台账相衔接、相对应、可追溯。广泛征求群众意见，科学编制“十三五”脱贫攻坚规划。

　　大力实施扶贫工程。抓紧实施扶贫攻坚专项方案，确保11.8万贫困人口脱贫，南部县、蓬安县实现脱贫“摘帽”。突出抓好产业培育，因地制宜扶持发展特色优势产业，健全新型经营主体、龙头企业、致富能人与贫困户利益链接机制；扎实抓好交通、水利、电力、信息等基础设施建设，大力改善生产生活条件，确保年内85%的贫困村通硬化路、农村集中供水率达到85%，完成所有贫困村电网升级改造；实施287个扶贫新村建设，完成12506户建档立卡贫困户的危房改造；切实抓好“零转移家庭”贫困劳动力和新生劳动力的培训转移，着力提高贫困群众自我发展能力；坚持教育、文化、医疗等公共服务资源向贫困地区倾斜，努力提高贫困群众社会保障水平。

　　加大扶贫投入力度。全力争取国省专项扶贫资金支持，积极争取金融扶贫、移民避险解困等试点项目。落实县级财政扶贫专项专户资金预算，确保在2015年的基础上增长20%以上；健全用扶贫资金捆绑其它涉农资金的机制，提高资金使用效益。严格落实财政扶贫资金项目公告公示制度，加大审计稽察力度，确保扶贫资金使用规范有效。积极开展定点帮扶、结对帮扶等活动，创新开展社会扶贫工作，广泛引导社会力量共同参与扶贫开发。

　　（六）加强社会建设，切实提高群众生活质量

　　全力办好民生大事实事。切实加大民生投入力度，确保财政投入用于民生支出比重稳定在70%以上。大力实施“十项民生工程”，突出抓好20件民生大事。实施更加积极高效的就业战略，确保新增城镇就业6万人以上。实施全民参保计划，推动机关事业单位养老保险制度改革，抓好城镇个体工商户、私营企业参保工作；启动实施失业保险市级统筹；全面开展大病医疗保险工作，全面落实医保市级统筹；持续提高城乡低保救助标准，保障困难群众基本生活。

　　不断强化基本公共服务。大力推进城乡义务教育均衡发展，切实抓好教育信息化建设；推动高中阶段教育逐步普及，加快建设现代职业教育体系，继续办好特殊教育和学前教育，启动南充国际高中、南充职业技术学院二期等重点项目建设，支持西南石油大学（南充校区）建设高水平工科大学。有序推进全民健身活动中心等项目建设，务实举办第五届市运会、第十八届中国老年合唱艺术节等重要文体活动。加快建设市中心医院江东院区、川北医学院附院新院区后续项目，启动建设川北医学院附属嘉陵医院、市中医院新院区。认真落实全面两孩政策。加快推进市规划展览馆、博物馆、文化馆、档案馆、地方志馆、南充大木偶剧院、川剧演艺中心建设；加大县、乡“两馆一站”建设力度，确保国家公共文化服务体系示范区创建成功。

　　切实提高社会治理水平。坚持系统治理、依法治理、综合治理、源头治理，不断完善社会治理体系，努力提高社会治理能力。扩大社会参与，支持社会组织在社会治理中发挥更大作用。畅通和规范群众诉求表达渠道，建立完善网上受理信访制度，切实解决群众合理诉求。层层压实安全生产责任，健全隐患排查治理和安全预防控制体系，深入开展重点行业领域安全专项治理，坚决遏制重特大安全生产事故发生。扎实推进食品药品安全监管信息化和检验检测体系建设，积极创建食品安全放心城市。深入推进“平安南充”建设，加快创新完善社会治安防控体系，加强反恐防暴工作，严厉打击各类违法犯罪行为，不断提高人民群众安全感。

四、加强政府自身建设

　　各位代表，蓝图已经绘就，使命催人奋进。面对新形势新常态，我们必须深入践行“三严三实”，严守党的政治纪律和政治规矩，切实增强政治意识、大局意识、核心意识、看齐意识。坚持以提高政府治理能力为导向，加强政府自身建设，全力迎接改革创新、转型发展的重大考验，全力迎接重塑形象、重振雄风的特殊检阅。

　　（一）深入推进民主施政。按照“公开为常态、不公开为例外”的原则，健全政府信息公开制度，全面推行行政决策公开，扩大市政府全体会、常务会、专家论证会、决策评估会的社会参与度，完善重大行政决策公众咨询制度。积极探索“互联网+交易”模式，推动公共资源交易平台从依托有形场所向以电子化平台为主转变，进一步提升行政审批、财政资金使用、公共资源配置、价格和收费信息等重点领域的公开度，让群众更好地监督政府。

　　（二）切实强化责任担当。坚持积极作为、主动作为、大胆作为，敢于担当、勇于担当、善于担当，切实做到守土有责、守土尽责、守土负责。深入推行部门责任公开承诺制，着力构建逐级抓落实的责任体系。坚持挂图作战，倒逼加压，确保重要决策、重大项目、重点工作落实落地。持续推行社会公众满意度测评，强化对政策执行、工作落实的监督检查。坚决整治庸政懒政怠政行为，让实干成为政府的作风导向，让担当成为干部的自觉追求。

　　（三）着力提高行政效能。大力简政放权，健全完善行政权力清单，全面推行政府责任清单，抓紧制定投资负面清单。深化商事制度改革，推行“三证合一”登记制度，实行电子营业执照和全程电子化登记。全面推行省市县三级便民联动审批，探索推行“互联网+政务服务”新模式，逐步实现网上申请、网上受理和网上审批。完善“12345”市民服务热线功能，打造政府高效服务新平台。

　　（四）依法规范权力运行。坚持在法治轨道上开展工作，依法全面履行政府职能，自觉接受市人大及其常委会的法律监督、工作监督和市政协的民主监督。健全依法科学民主决策机制，全面推行权力运行网上公开，充分发挥政府法制机构、法律顾问团的作用，努力提高决策质量。深化行政执法体制改革，积极探索跨部门、跨行业综合执法，切实做到严格规范公正文明执法。

　　（五）全力重塑政务形象。始终把廉洁从政作为政府工作的钢要求、铁纪律，坚决贯彻从严治党、依法行政各项规定，严格落实市委“三个带头、四个管好”的要求，切实履行党风廉政建设责任制，深入推进反腐倡廉。着力推动党风廉政建设向基层延伸，坚决纠正侵害群众利益的不正之风。强化制度建设，切实做到用权有依据，行权有出处，笼子扎得紧。严格执行中央“八项规定”和省委省政府“十项规定”，坚决防止“四风”反弹，树立清正廉洁的良好形象。

　　各位代表！富民强市的梦想激荡，全面小康的步伐铿锵。让我们更加紧密地团结在以习近平同志为总书记的党中央周围，在省委省政府和市委的坚强领导下，紧紧依靠和团结全市人民，以逢山开路、遇水架桥的闯劲，以踏石留印、抓铁有痕的韧劲，以甩开膀子、迈开步子的干劲，奋力拼搏，砥砺前行，为加快建设创新、开放、生态、法治、繁荣的区域中心城市而努力奋斗！
